# Association between malaria and malnutrition among children aged under-five years in Adami Tulu District, south-central Ethiopia: a case–control study

**DOI:** 10.1186/s12889-016-2838-y

**Published:** 2016-02-19

**Authors:** Bilal Shikur, Wakgari Deressa, Bernt Lindtjørn

**Affiliations:** Department of Reproductive Health and Health Service Management, School of Public Health, College of Health Sciences, Addis Ababa University, Addis Ababa, Ethiopia; Department of Preventive Medicine, School of Public Health, College of Health Sciences, Addis Ababa University, Addis Ababa, Ethiopia; Centre for International Health, University of Bergen, Bergen, Norway

**Keywords:** Ethiopia, Malaria, Malnutrition, Case-control

## Abstract

**Background:**

Malaria and malnutrition are the major causes of morbidity and mortality in under-five children in developing countries such as Ethiopia. Malnutrition is the associated cause for about half of the deaths that occur among under-five children in developing countries. However, the relationship between malnutrition and malaria is controversial still, and it has also not been well documented in Ethiopia. The aim of this study was to assess whether malnutrition is associated with malaria among under-five children.

**Methods:**

A case–control study was conducted in Adami Tulu District of East Shewa Zone in Oromia Regional State, Ethiopia. Cases were all under-five children who are diagnosed with malaria at health posts and health centres. The diagnosis was made using either rapid diagnostic tests or microscopy. Controls were apparently healthy under-five children recruited from the community where cases resided. The selection of the controls was based on World Health Organization (WHO) cluster sampling method. A total of 428 children were included. Mothers/caretakers of under-five children were interviewed using pre-tested structured questionnaire prepared for this purpose. The nutritional status of the children was assessed using an anthropometric method and analyzed using WHO Anthro software. A multivariate logistic analysis model was used to determine predictors of malaria.

**Results:**

Four hundred twenty eight under-five children comprising 107 cases and 321 controls were included in this study. Prevalence of wasting was higher among cases (17.8 %) than the controls (9.3 %). Similarly, the prevalence of stunting was 50.5 % and 45.2 % among cases and controls, respectively. Severe wasting [Adjusted Odds Ratio (AOR) =2.9, 95 % CI (1.14, 7.61)] and caretakers who had no education [AOR = 3, 95 % CI (1.27, 7.10)] were independently associated with malarial attack among under-five children.

**Conclusion:**

Children who were severely wasted and had uneducated caretakers had higher odds of malarial attack. Therefore, special attention should be given for severely wasted children in the prevention and control of malaria.

## Background

Malaria is one of the major health problems in sub-Saharan Africa. According to the 2013 world malaria report, malaria is a cause for about 627,000 malaria deaths worldwide. The report also indicates that 3.4 billion people were at risk of malaria. Most of these malaria deaths (90 %) occurred in sub-Saharan Africa and in children under five years of age [1].

On the other hand, malnutrition is the underlying cause of over 50 % of child deaths in developing countries [2]. Malnutrition and infection interrelate synergetically [3]. In other words, malnutrition increases susceptibility to infection while episodes of infection, in turn, precipitate nutritional deficiencies. Ethiopia has a high prevalence of malnutrition in children. Based on the 2011 Ethiopian Demographic and Health Survey (EDHS), the prevalence of stunting, being underweight and wasting among under-five children are 44 %, 29 % and 10 % respectively [4].

Research evidence shows that malnourished children have lower anti-malarial immunity. For instance, a study carried out by Fillol showed that both the prevalence of anti-malarial immune respondents and specific IgG Ab levels were significantly lower in malnourished children than in children who were not malnourished [5]. In the same study, wasted children and stunted children presented a lower specific IgG Ab response than their respective controls. Similarly, a study conducted in *Papua* New Guinea revealed that humeral responses to specific malarial antigens were the lowest in the wasted children [6].

Even though there are evidences that showed malnourished children have lower anti-malarial immunity, there are contradictory findings on the association between malaria and malnutrition [6–8]. Some studies showed increased risk of malaria attack among malnourished children. For instance, prospective cohort studies in rural Gambia and Uganda reported children with stunting had a higher incidence of malaria compared to children without stunting [7, 8]. In addition, another study in Nigeria revealed positive relationship between severe acute malnutrition and malaria [9]. In the contrary to the above findings, a cohort study in Papua New Guinea indicated that as height for age z-score (HAZ) increases, the risk of *P. falciparum* malaria attacks also increase [6]. On the other hand, no association between malnutrition (underweight, stunting, wasting) and malaria was reported in the study carried out in south-west Ethiopia [10]. Absence of association in this study could perhaps be due to the difference in the definition given to malaria. In this study, unlike the other studies in which the definition of malaria is based on fever and parasitemia, malaria is defined based only on parasitemia.

A variety of intervention strategies have been used to combat malaria and this has led to a significant decline in mortality in Ethiopia. Long Lasting Insecticidal Nets (LLIN), chemoprophylaxis and prompt and appropriate case management can be mentioned as major examples of intervention strategies [11]. No single strategy or solution has so far been successful in the fight against malaria; instead, a comprehensive approach is required. Therefore, identifying further predisposing conditions for malaria such as malnutrition may broaden available intervention strategies to combat malaria. However, the relationship between malnutrition and malaria is controversial still. This study aimed at generating evidence on whether malnutrition, particularly, wasting, stunting and underweight, is associated with malaria.

## Methods

### Study area and population

An un-matched case–control study was conducted among under-five children during February-April 2014 in Adami Tulu district which is located 160kms south of Addis Ababa. The district lies at an altitude between 1,500 and 1,750 m above sea level. According to the 2007 national census, the population was about 150,000 of which 17 % were under-five children [12]. The district has one public hospital, nine health centres, and 43 rural health posts.

The transmission of malaria in the district is seasonal. Major transmission of malaria occurs from September to December; following the summer rainfall. *P.falciparum and P.vivax* are the most common malaria parasites in the district. *Anopheles arabiensis* is considered as the main vector [11]*.*

Malnutrition is a very common problem in the region. The prevalence of stunting, being underweight and wasting are 41 %, 26 % and 9.7 % respectively [4]. Crop production is the major economic activity followed by livestock rearing. Maize is the main crop produced and consumed by the inhabitants. November and December are harvesting months. The district is characterized by high burden of hunger in the months of July and August.

The study population included cases and controls from selected health facilities and community, respectively. Cases consist of children aged between 6–59 months who visited the selected health facilities and diagnosed as malaria positive. The diagnosis was confirmed by microscopy in the health centres and Rapid Diagnostic Test (RDT) in health posts during data collection period. Controls include under-five children aged 6–59 months who were apparently healthy and lived in the community where cases resided. Controls are considered to be apparently healthy if the mother/caretakers perceived the child to be healthy and declared no symptoms of disease such as fever, cough, difficulty of breathing, and diarrhoea. However, these were not confirmed by laboratory. More than 90 % of cases came from the same kebeles (community) of controls. Kebeles are categorized as urban if the population density is greater than 150 people per km^2^. Otherwise, they are considered as rural.

### Sample size and sampling procedure

Sample size was calculated using Epi-Info Statcalc version 3.5.4. The following assumption was made during the calculation: 95 % confidence interval, 80 % power, 10 % wasting in controls, Odds Ratio (OR) =2.50 [13], ratio of case to controls 1:3. Adding 5 % non-response rate in to these assumptions made the sample size to be 441 (110 cases and 331 controls). A total of nine health facilities were purposively selected because of their high malaria patient flow in the year prior to the study year. These include three health centres and six health posts. All confirmed malaria cases, from the selected health facilities, were included until the required sample size was achieved.

On the other hand, controls were randomly selected from four kebeles( the lowest administrative unit) where cases were identified. Sampling of controls was done using sampling technique of World Health Organization (WHO) Expanded Program of Immunization (EPI) cluster sampling method [14]. First, equal size of controls was allocated to each of the four selected kebeles. Then a random direction was chosen from the midpoint of each selected kebeles and starting from the first dwelling in each kebele, interviewers moved from house to house in a predetermined manner stopping at a house where allocated sample size was achieved . All children aged 6–59 month were included in each selected household.

### Data collection and quality assurance

A two-day long training was given to nine data collectors and one supervisor. The training focused on the questionnaire and anthropometric measurements. The questionnaire comprised of socio-demographic characteristics of children and their care takers/mothers such as sex of the child, age of the child and mother, mean monthly family income, educational status, occupation, marital status and ethnicity of the mother. Questions on malaria prevention practice like use of LLIN and Indoor Residual Spray (IRS), anthropometry measurement and laboratory test for malaria were also included. Both open ended and closed ended questions were used. In average the interview took 30–40 min. Data collection instruments were pretested and validated.

For suspected malaria cases in the health centres and health posts, blood was taken from finger prick to confirm malaria. Laboratory technicians who had at least two years of work experience at selected health centres prepared thick and thin blood films. The technicians used standard operating procedures to do the microscopy. Multi-species RDT kit was used by health extension workers (HEWs) using the Ministry of Health malaria diagnosis and treatment guideline at health posts [11]. Regular supervisions were made by the supervisor and principal investigator.

Weight of the children was measured using a digital weighing scale in bare foot and with light clothing. Height was measured for children above two years of age while length for children below two years of age using length/height board. The measurement was rounded to the nearest 0.1 kg and 0.1 cm for the weight and height respectively. Weighing scale was calibrated frequently. The accuracy of weighing scale was checked by placing a weight of 2 kg iron bars on the scale. A local calendar was prepared to improve accuracy in estimating children’s age.

### Data processing and analysis

Data were entered using Epi-Info version 3.5.4, then exported to SPSS version 21 for cleaning and analysis. For the analysis of nutritional indices the data were exported to WHO Anthro software. The WHO 2006 growth standards were used to transform children’s weight and length/height measurements into sex- and age-specific Z-scores. Children were classified as stunted, wasted, and under-weight if the HAZ, WHZ, WAZ < −2 standard deviation (SD) respectively. They were categorized as severely stunted, severely wasted or severely underweight if the HAZ, WHZ, WAZ were < −3 SD, respectively.

Data were summarized and presented in frequency table. Associations between independent variables and outcomes of interest were estimated using both bivariate and multivariate logistic regression. Independent variables with a *P*-value <0.2 in bivariate analyses were included for multivariate analysis. Final multivariate model was generated using enter method. In the final interpretation of results a *P*-value <0.05 was considered statistically significant. The adequacy of our model was tested using Hosmer-Lameshow goodness of fit test and it was found to be worthwhile (*P* = 0.57).

### Ethical consideration

The proposal was reviewed and approved by the research and ethics committee of the School of Public Health, Addis Ababa University. Written informed consent was obtained from all mothers/ caretakers who participated in the study. This was done following explanation of the purpose of the study to the participants. Treatment was provided to children with confirmed malaria according to the national guideline in the respective health centres and health posts. Severely malnourished children were also linked to the therapeutic feeding centres.

## Results

### Background characteristics of caretakers

From a total estimated sample size of 441, 428 mothers/caretakers of children were interviewed (making response rate of 97 %) of which 25 % (*n* = 107) were from cases and 75 % (*n* = 321) were from controls. As shown in Table 1, 76.6 % (*n* = 82) of the caretakers of the cases and 77 % (*n* = 247) caretakers of controls were rural residents. Majority of the caretakers of both cases 65.4 % (*n* = 70) and controls 56.4 % (*n* = 181) had no education.

**Table 1:** Background characteristics of mothers/caretakers of under five children, Adami Tulu district, Ethiopia 2014 (*N* = 428)

Caretakers	Cases	Controls	*P*-value
Characteristic	N (%)	N (%)	
Sex			
Male	25 (23.36 %)	66 (20.56 %)	0.54
Female	82 (76.6 %)	255 (79.4 %)	
Mean age (years	29 (SD = 6.90)	28 (SD = 7.70)	0.15
Residence			
Rural	82 (76.63 %)	247 (76.95 %)	0.94
Urban	25 (23.36 %)	74 (23.05 %)	
Ethnicity			
Oromo	98 (91.59 %)	296 (92.21 %)	0.83
Others	9 (8.41 %)	25 (7.78 %)	
Educational status			
No education	70 (65.42)	181 (56.39 %)	0.03
Primary (grade 1–8)	29 (27.73 %)	89 (27.72 %)	0.09
≥ Secondary (≥9)	8 (7.48 %)	51 (15.89 %)	
Occupation			
Housewife	70 (65.42)	193 (60.13 %)	0.61
Farmer	27 (25.24 %)	96 (29.91 %)	0.98
Daily labourer	5 (4.67 %)	14 (4.36 %)	0.73
Other	5 (4.67 %)	18 (5.61 %)	
Marital status			
Married	104 (97.20 %)	304 (94.70 %)	0.30
Others	3 (2.80 %)	17 (5.30 %)	
Mean Monthly family			
income(ETH birr)	744 (SD = 603)	678(SD = 682)	0.34
Radio			
Yes	58 (54.20 %)	201 (62.62 %)	0.12
No	49 (45.80 %)	120 (37.38 %)	
Roof			
Thatched	37 (34.58 %)	84 (26.20 %)	0.09
Iron	70 (65.42 %)	237 (73.83 %)	
Bicycle			
Yes	26 (24.30 %)	84 (26.20 %)	0.70
No	81 (75.70 %)	237 (73.83 %)	

### Comparison of nutritional status and other features between cases and controls: Bivariate analysis

As indicated in Table 2, 65.4 % (*n* = 70) of cases and 55.8 % (*n* = 179) of controls were males. The mean age of children was 28 (SD ± 14) months for cases and 33 (SD ± 16) months for controls. Prevalence of wasting was higher among cases 17.7 % (*n* = 19) than controls 9.3 % (*n* = 30). High proportions of cases were severely wasted 13.1 % (*n* = 14) than the controls 3.7 % (*n* = 12). There was no significant difference in the prevalence of wasting among cases diagnosed in health centres or health posts. Prevalence of underweight was higher in cases 24.3 % (*n* = 26) than in controls 18.4 % (*n* = 59) and 13.1 % (*n* = 14) of the cases and 5.3 % (*n* = 17) of the controls were severely underweight.

**Table 2:** Comparison of nutritional status and other features between cases and controls Adami Tulu district: Bivariate analysis 2014 (*N* = 428)

Variables	Cases	Controls	*P*-value
	N (%)	N (%)	
Sex			
Male	70 (65.42 %)	179 (55.76 %)	0.08
female	37 (34.58 %)	142 (44.24 %)	
Age in months			
6–24	50 (46.72 %)	116 (36.13 %)	0.05
≥25	57 (53.27 %)	205 (63.86 %)	
Severe wasting (WHZ < − 3)	14 (13.08 %)	12 (3.74 %)	0.01
Moderate wasting ( −3 ≤ WHZ < −2)	5 (4.67 %)	18 (5.61 %)	0.87
No-wasting (WHZ ≥ −2)	88 (82.24 %)	291 (90.65 %)	
Severe stunting ( HAZ < −3)	29 (27.10 %)	78 (24.30 %)	0.43
Moderate stunting ( −3 ≤ HAZ < −2)	25 (23.36 %)	67 (20.87 %)	0.45
No-stunting (HAZ ≥ −2)	53 (49.53)	176 (54.83 %)	
Severe underweight (WAZ < −3)	14 (13.10 %)	17 (5.30 %)	0.01
Moderate underweight (−3 ≤ WAZ < −2)	12 (11.21 %)	42 (13.10 %)	0.82
No underweight (WAZ ≥ −2)	81 (75.70 %)	262 (81.62 %)	

About half of the malaria cases 50.5 % (*n* = 54) were detected in health centres using microscope and the rest 49.5 % (*n* = 53) were diagnosed in health posts using RDT. One quarter (*n* = 27) of cases and about two fifth (*n* = 123) of controls used LLIN the night before the interview. Regarding indoor residual spraying, 66.3 % (*n* = 71) of the cases and 73.2 % (*n* = 235) of the controls households had been sprayed within the past one year.

### Nutritional and other factors associated with malaria among under-five children

As shown in Table 2, severe wasting (*P*-value = 0.01), being severely underweight (*P*-value = 0.01), sleeping under LLIN of the child the night before the study (*P*-value = 0.015), educational status of caretakers (*P*-value = 0.026) were significantly associated with malaria attack in children in bivariate logistic regression. Severe wasting and educational status of the caretakers remained significant after adjusting for the following confounders using multivariate logistic model: age and sex of the child, religion, LLIN utilization of the child and IRS. WAZ was reduced from the model because there was multi-collinearity among anthropometric indicators.

As indicated in Table 3, severely wasted children (WHZ < −3) were 3 times more likely to have malarial attack than non-wasted children (adjusted OR = 2.9, 95 % CI: 1.14, 7.61, *P*-value: 0.025). The dose response trend between wasting and malaria was analyzed using Mantel-Haenszel chi square for linear trend and it was found to be significant (*p* = 0.003). Children with caretakers who had no formal education were 3 times more likely to have malarial attack than those with secondary education or above (adjusted OR = 3, 95 % CI: 1.27, 7.00).

**Table 3:** Nutritional and other factors associated with of malaria in under-five children, Adami Tulu district, Ethiopia 2014 (*N* = 428)

Factors	Cases	Controls	Crude OR	Adjusted OR	95 % CI	
					Lower	Upper
Child age (months)						
6–24	50	116	1.55	1.55	0.96	2.51
25–59	57	205	1.00	1.00		
Sex						
Male	70	179	1.55	1.51	0.93	2.47
Female	37	142	1.00	1.00		
Sleep under ITN last						
night						
Yes	27	123	0.54	0.65	0.38	1.11
No	80	198	1.00	1.00		
Literacy of respondent						
No education	70	181	2.46	*2.99* ^a^	1.27	7.61
Primary(1–8)	29	89	2.08	2.41	0.96	6.06
Secondary(9–12)	8	51	1.00	1.00		
Wasting						
Severe wasting (WHZ < − 3)	14	12	3.86	*2.95* ^a^	1.14	7.60
Moderate wasting ( −3 ≤ WHZ < 2)	5	18	0.92	0.66	0.21	2.03
No-wasting (WHZ ≥ −2)	88	291	1.00	1.00		
Indoor residual						
Spraying						
Yes	71	235	0.72	0.64	0.37	1.11
No	36	86	1.00	1.00		

^a^Indicates significant OR

## Discussion

This study compared the prevalence of malnutrition in under-five children between malaria cases and controls so as to see the association between malnutrition and malaria. After controlling for possible confounders there was a significant positive association between wasting and malaria in under-five children. Severely wasted children were 3 times more likely to have malarial attack than non-wasted children.

The increased odds of malarial attack in severely wasted children compared to the non-wasted ones can be explained by the immunosuppressive effect of severe wasting. Our study is consistent with a case control study done in Nigeria which revealed high likelihood of malaria in acutely malnourished children than in healthy ones [9]. Another study conducted in Democratic Republic of Congo also revealed that children with severe protein energy malnutrition were at higher risk of clinical malaria [15]. Similarly, a cross sectional study done in Rwanda showed that children with low mid upper arm circumference (MUAC) were significantly associated with increased *P.falciparum* infection [16].

A retrospective analysis of routine programme data on forty-eight nutritional rehabilitation centres in southern Ethiopia showed that 28 % of malnourished children with fever (Temperature >37.5 °C) were positive for malaria on RDT. Even though this study did not compare malaria in malnourished and well nourished children, the study revealed a significant positive association between malaria prevalence and increasing grades of malnutrition which is consistent with our finding [17].

In contrast to the finding noted in the present study, a cohort study carried out in rural preschool children in Senegal revealed that wasted children had lower risk of having malaria attack [5]. However, in this study the prevalence of wasting was relatively low (3.10 %). In addition, a study done in south west Ethiopia revealed no association between wasting and malaria [10]. In this study, although the total sample size was large, only 3.10 %( *n* = 7) of wasted children were positive for malaria. Other than what we mentioned above, the definition of malaria in the above study was solely based on parasitemia unlike our study which is based on both fever and parasitemia.

The attempt made to understand the association between malnutrition and infection in general enhances our understanding the association between malnutrition and malaria. Malnutrition increases susceptibility to infection. On the other hand, infection has adverse effect on nutritional status. The reduced resistance to infection in malnourished children is mainly explained by the suppression of their immunity. Malnutrition affects not only the humeral and cell mediated immunity but it also affects bactericidal activity of phagocytes and complement formation [3]. There is evidence to support the hypothesis that malnourished children have lower immunity against malaria parasite. A study in Papua New Guinea revealed that humoral responses to malarial antigens were significantly lower in wasted children than in normal ones. The same study showed wasted children had more malarial attack than well-nourished children; however the difference was not significant [6]. Another study in Senegal has also revealed a lower level of specific immunoglobulin-G (IgG) response in malnourished children than in the controls [5].

The association between malnutrition and malaria is controversial [5, 6, 9]. The difference in study design could be a possible explanation for the conflicting findings. When studies compare nutritional status in malaria cases and controls, it’s important to carefully select the controls. For instance, it is known that common childhood illnesses such as pneumonia and diarrhoea are highly associated with malnutrition. If such sick children are included in the control group to compare them with malaria cases, this will distort the true association between malaria & malnutrition. In such studies if malnutrition is found to be less in malaria patients, compared to the comparison group, we cannot conclude that malnutrition is protective against malaria. Instead we can probably say malnourished individuals are at higher risk of diseases like pneumonia or diarrhoea than that of malaria. Therefore having healthy comparison group is a very important issue to consider when we want to see the relationship between malaria and malnutrition.

Another important reason for conflicting findings can probably be due to the difference in the case definition of malaria. In other words, some studies defined malaria based on either parasitemia or fever while others based on both fever and parasitemia. Although fever is the most important symptom of malaria, it is not unique for malaria [18]. On the other hand, malnourished children are at a higher risk of infection but they may not have fever when infected because of poor immune response [19]. Malaria parasitemia could be low in severely malnourished individuals as a result of poor multiplication of the parasite due to lack of nutrients essential for the multiplication or due to adverse environment in the human body associated with production of toxins in severely malnourished individual. However severely malnourished children may not be able to clear the infection because of suppressed immunity. Relapse of malaria can probably occur when conditions are favourable for the parasite [20]. Therefore, in this study the case definition for malaria included both fever and parasitemia which is in line with the national malaria guideline [11].

A further possible explanation for the controversy could be the fact that many studies, including the present study, do not control the effect of specific micronutrients deficiency on malaria morbidity. Micronutrient deficiency is as important as protein energy malnutrition which usually occurs together. This is to say, micronutrient deficiency determines how malnutrition is associated with malaria [18].

In conducting case–control studies it’s crucial to minimize selection and recall bias. In this study, since exposure status was measured objectively using anthropometry recall bias is believed to be low. Comparability between cases and controls is another major concern that can affect the association between the exposure and outcome of interest. In our case, controls were randomly selected from the source population of cases which is very important to ensure the comparability.

As a result of high awareness about malaria in the district (Part of the finding of this study but not presented in this paper) and proximity of the health facilities, caretakers probably brought their children within few days of occurrence of fever to the nearby health facility. Therefore, it was less likely that malaria could result in such level of malnutrition within in short period of time. This is to say that malnutrition may possibly be a risk factor for malaria. However, we can’t confirm the temporal relationship between malaria and malnutrition in this study since malaria can cause malnutrition and the vice versa.

In our study, we did not include children admitted to hospitals or health centers for severe acute malnutrition. This could have an effect on our findings of the association between malaria and malnutrition. The effect however, depends on several factors like whether those children admitted for severe acute malnutrition (SAM) have super infections with pneumonia or other childhood infections. In this case, the association between malaria and malnutrition could not be affected since such children would have been ineligible to be either cases or controls in our study. On the other hand, if large proportions of children admitted with SAM have malaria, these would have been potential cases. Hence, our finding might have underestimated the actual Odds Ratio for malaria and malnutrition. Conversely, if large proportion of children were admitted for SAM without superimposed infection or malaria, they would have been potential controls. Consequently, our finding might have overestimated the actual Odds Ratio for malaria and malnutrition.

One of the strengths of this study is the classification of malnutrition based on the degree of severity. The finding of higher odds of malarial attack among severely wasted children, but not moderately wasted ones, could be explained by the higher level of immunosuppression among severely wasted children compared to moderately wasted ones.

Caretaker’s/mothers lack of education is another most important factor that was positively associated with under-five malaria attack. This is in line with studies done in rural Rwanda and Tanzania [16, 21].

However this study had some limitations. The effect of micronutrients on malaria morbidity was not explored. In addition, there are also possible confounding factors such as diarrhoea, vomiting, and source of water supply that were not included in this study. Severely malnourished children who were admitted for different reason were not also included in this study. Furthermore non-random selection of cases might affect the generalizability of the study. We have recruited controls from the community because it was difficult to get apparently healthy children in the health facilities. However, the fact that we used different base populations for the cases (health facility) and controls (community) can be considered as another limitation of this study.

## Conclusion

This study revealed severe wasting and maternal education as independent predictors of malaria. Severely wasted under-five children were more likely to develop malarial attack than non-wasted children. Children with uneducated mothers/care takers were also more likely to develop malaria.

### Recommendations

Therefore, special attention should be given for severely wasted children the prevention and control of malaria. In addition, education of girls should be supported. Further research on the effects of micronutrients on malarial morbidity should be done.
